# Health Habits and Wearable Activity Tracker Devices: Analytical Cross-Sectional Study

**DOI:** 10.3390/s22082960

**Published:** 2022-04-12

**Authors:** Héctor José Tricás-Vidal, María Orosia Lucha-López, César Hidalgo-García, María Concepción Vidal-Peracho, Sofía Monti-Ballano, José Miguel Tricás-Moreno

**Affiliations:** 1 Unidad de Investigación en Fisioterapia, Universidad de Zaragoza, Domingo Miral, s/n, 50009 Zaragoza, Spain; tricasdpt@gmail.com (H.J.T.-V.); cvidal@unizar.es (M.C.V.-P.); smonti1395@gmail.com (S.M.-B.); jmtricas@unizar.es (J.M.T.-M.); 2 School of Health Professions, University of Mary Hardin Baylor, 900 College St., Belton, TX 76513, USA; 3 Department of Endocrinology and Nutrition, Hospital Royo Villanova, SALUD, Barrio San Gregorio s/n, 50015 Zaragoza, Spain

**Keywords:** wearable activity tracker, BMI, physical activity

## Abstract

Wearable activity trackers are electronic devices that facilitate self-monitoring of information related to health. The purpose of this study was to examine the use of tracker devices to record daily activity (calories) and its associations with gender, generation, BMI, and physical activity behavior of United States of America resident adults; a cross-sectional study in 892 subjects recruited to participate in an anonymous online survey was performed. Being female increased the odds of using a tracker device by 2.3 times. Having low cardiovascular disease mortality risk related to time spent sitting increased the odds for using a tracker device by 2.7 times, and having medium risk 1.9 times, with respect to having high risk. For every 1-point increase in BMI, the odds for using a tracker device increased by 5.2%. Conclusions: Subjects who had ever used any tracker device had a higher BMI. The use of tracker devices was related to lower cardiovascular disease mortality risk related to sitting time. The amount of physical activity and the time spent walking were not associated with the usage of tracker devices. It is possible that the user of tracker devices should be supported by professionals to implement deep change in health habits.

## 1. Introduction

Wearable activity trackers (for example, Fitbit, Apple Watch, Polar, Garmin, or Nike FuelBand) are electronic devices that facilitate self-monitoring and tracking of activities and information related to fitness or physical activity [1]. The use of wearable activity tracker devices has increased exponentially in the last decade [2]. The volume of shipments of these devices reached 72.6 million units according to data from the International Data Corporation Worldwide Quarterly Wearable Device Tracker Global, during the first quarter of 2020; thus, wearable devices shipments grew 29.7% year over year [2]. The most sold devices in May 2020 were Apple, Xiaomi, Samsung, Huawei, and Fitbit [2]. In a Pew Research Center survey conducted on 3–17 June 2019, one in five United States of America (US) adults (21%) said they regularly carried a smartwatch or wearable activity tracker [3]. One in three Americans reported having had a wearable activity tracker such as a Fitbit or smartwatch (34%) according to the global analytics and advice firm Gallup, in 1–14 November 2019 [4].

Wearable activity trackers allow monitoring of daily activity, such as steps taken, timing and intensity of physical activity, distance covered, calories burned, active time, sleep assessment [5], and heart rate [6], and may include mobile connectivity or an internet application. Due to the characteristics of the information they collect, they can be an exhaustive source of information on the health habits of the population that can be shared with health professionals and serve as a diagnostic tool on the quantity and quality of physical activity as well as other health-related habits such as the quantity and quality of sleep. However, most of the time, the population uses wearable activity tracker devices, but does not necessarily share the information obtained with professionals who can help them with interpretation and making it effective [7]. Sharing data from wearable devices with healthcare professionals would allow them to better understand their patients’ habits [7].

Behind the increased use of wearable activity tracker devices are a diversity of factors: self-determination [8], self-awareness, motivation, tracking progress, and staying informed [9]. Wearable activity tracker devices have been shown to increase exercise motivation through different constructs [10]. Some constructs are related to social interactions [10]. Others are in relation with exercise control features and with data management features as possibilities for data analysis, data collection, progress updates [10], or constructive feedback [8]. In young populations, the importance of daily sociability, psychological factors such as high extroversion levels, and behavioral factors such as large network size have been established as modulators of physical activity implication [11]. It has been observed that personalized feedback facilitates positive emotional responses for highly active participants. Subjects with low activity may experience negative emotional responses but also positive coping mechanisms [12].

Activity trackers have shown the potential to increase physical activity, but the effects on weight loss remain contradictory [13]. Moreover, the use of tracker devices to record daily activity (calories) has been related to the possibility to trigger, maintain, or exacerbate eating disorders [14]. Previous studies have focused on the adoption of technology products in different age ranges [15,16,17,18,19], but research on the differences in the adoption of wearable activity tracker devices between generations is sparse. For example, regarding the use of wearables in adolescents, the literature shows contradictory results [20,21]. Moreover, there are still open questions regarding tracker device use differences across genders. It has been shown that more women have participated in studies about the efficacy of wearable activity devices when used in a comprehensive weight loss program [22]. It is also necessary to consider that wearable activity tracker devices may function inaccurately [23]. The need to adapt the type of device to the characteristics of the users has also been discussed and it has been seen that it is important for long-term use, to facilitate the user experience in terms of functionalities and aesthetics and physical design [24]. The controversial data regarding the effectiveness of tracker devices to record daily activity to promote healthy habits suggests increasing the available evidence on its use.

There are few studies that relate the use of wearable activity tracker devices to the general population in terms of health habits, such as healthy weight maintenance and physical activity behavior. The purpose of this study was to examine the use of tracker devices to record daily activity (calories) and its associations with gender, generation, BMI, and physical activity behavior of US resident adults.

The manuscript presents in the material and methods section the methodology used to collect and analyze the data. The results of the data analysis are presented below, in the results section. Then, in the discussion section, the results are discussed according to the available scientific evidence and finally the conclusions of the study are presented.

## 2. Materials and Methods

A non-experimental analytical cross-sectional study with multivariate analysis was performed.

The Academic Commission of the Doctoral Program in Health and Sports Sciences of the University of Zaragoza approved the study, which complied with the ethical requirements of the Declaration of Helsinki [25].

### 2.1. Subjects

A cohort of 892 subjects was recruited. It consisted of US residents recruited via email to participate in an anonymous online survey.

An invitation via email account with the survey link was sent to former or current students from Queens University of Charlotte, The University of Kentucky, Oakland University, and the University of Mary Hardin Baylor. Moreover, the survey link was published on Instagram and Facebook and was connected to the Survey Monkey website. Diffusion of the link was carried out with a snowball effect. Answers were collected using that online platform for later analysis.

To calculate the sample size, we used the United States Population: 329,256,465 (July 2018 statistics) according to The World Factbook [26]. The expected proportion used was 21% because one-in-five US adults (21%) said they regularly carried a smartwatch or wearable fitness tracker [3]. The sample size was calculated using the GRANMO calculator [27], with the population estimation option, confidence level 0.95, with the desired precision of +/− 3 percent units. A minimum number of 709 subjects was obtained.

**Figure 1:**
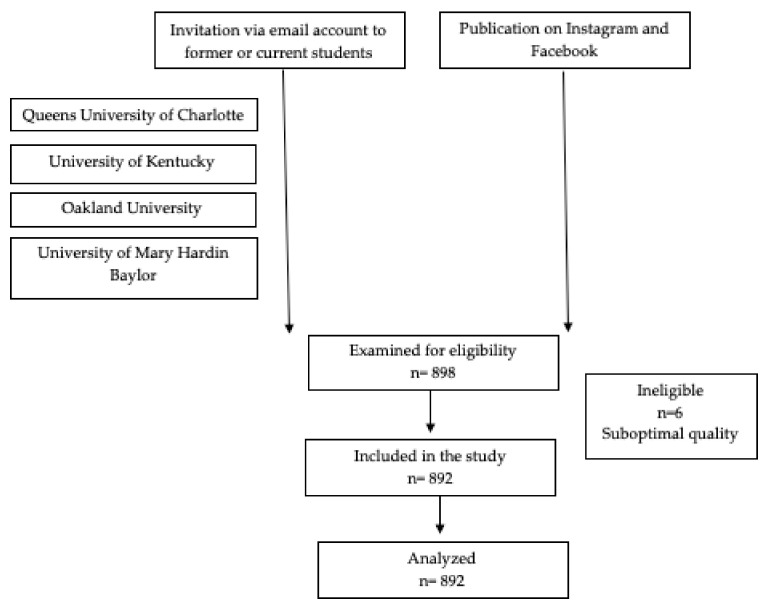
Flow chart of the survey sample.

Finally, 892 participants were analyzed, after the elimination of 6 surveys that inadequately answered the question related to tracker device use (Figure 1). Participants were required to be over 18 years old and they needed to have an Instagram account. None of the participants were compensated for participating in this research.

The study did not ask participants questions regarding race, sexual identity, religion, political views, or other questions that could break the law regarding research ethics. All subjects, after clicking on the link to be directed to the survey, provided consent.

### 2.2. Data Sources

The participants answered the following questions in the anonymous online survey (Table 1):

- Gender. Male/female.
- Age. Age was categorized according to the next generations: generation-Z (born 1997–2012); millennials (born 1981–1996); generation-X (born 1965–1980); boomers (born 1946–1964) [28].
- Height in feet and inches and weight in pounds. BMI was calculated: BMI = 703 × weight (pounds)/[height (inches)]^2^.
- Have you ever used any of the following tracker devices to record your daily activity (calories)? The possible options were: a. Fitbit, b. Apple Watch, c. Polar, d. Garmin, e. Nike, f. Other (please name), g. I have never used any tracking device. The question was categorized as “Ever used” (if a participant selected some option from a to f) or “never used” (if the participant selected the option g).
- Physical activity carried out by the participants was collected with the self-administered International Physical Activity Questionnaire (IPAQ) short form “last 7 days” [29]. It has been demonstrated that reliable and valid physical activity data can be collected with the IPAQ short form [30]. Vigorous physical activity (min per week), moderate physical activity (min per week), time spent walking (min per week), and time spent sitting (hours per day) were registered.

Time spent seating was recoded: low cardiovascular disease mortality risk indicated sitting less than 4 h per day; medium risk indicated sitting 4–8 h per day; high risk indicated sitting 8–11 h per day; very high risk indicated sitting more than 11 h per day [31].

**Table 1:** Data sources.

**Anonymous Online Survey**	
**Gender**	Male
	Female
**Generation**	Generation-Z (born 1997–2012)
	Millennials (born 1981–1996)
	Generation-X (born 1965–1980)
	Boomers (born 1946–1964)
**Body Mass Index**	703 × weight (pounds)/[height (inches)]^2^
**Use of tracker device to record daily activity (calories)**	Ever used
	Never used
**International Physical Activity Questionnaire short form “last 7 days”**	**Time spent sitting**
	Low cardiovascular disease mortality risk (sitting less than 4 h per day)
	Medium cardiovascular disease mortality risk (sitting 4–8 h per day)
	High cardiovascular disease mortality risk (sitting 8–11 h per day)
	Very High cardiovascular disease mortality risk (more than 11 h per day)
	**Vigorous physical activity (min per week)**
	**Moderate physical activity (min per week)**
	**Time spent walking (min per week)**

### 2.3. Statistical Analyses

The numerical analysis was performed using SPSS 25.0 for Mac. Statistical significance was set at *p* < 0.05.

A descriptive analysis of qualitative variables, offering the absolute frequencies, and the percentages in each category and of quantitative variables, offering the mean ± standard deviation was carried out.

To examine the relationship between variables, “use of tracker device to record daily activity (calories)” was established as the independent variable. If the dependent variable was qualitative, Chi-square was used (the maximum likelihood ratio Chi-square test was selected if the data set did not meet the sample size assumption of the Chi-square test) and if the dependent variable was quantitative the U-Mann–Whitney test was used.

To model the use of tracker devices to record daily activity (calories) as a function of the variables with significative relationships previously detected, one generalized linear model (GLM) was used. The model type used was main effects with Binomial as the distribution and Logit as the link function. The parameter estimation method was the hybrid method, and the scale parameter was Pearson chi-square.

Model assumptions were verified. The goodness of fit to check for under or over dispersion of the data was tested. The dispersion coefficient is the deviance value/degrees of freedom. In models with binomial distribution, it should give a value close to 1. If it is >1, there is over dispersion; if it is <1, it is said that there is under dispersion.

Distribution of the deviance residuals was tested using a probability plot. The residuals are the differences between the values estimated by the model and the observed values. In the case of binomial models, this graph shows a distribution in two lines, with antisymmetric distal extremes and proximity of both medial extremes.

Relationship between deviance residuals and model predictions was verified by plotting deviance residuals versus predicted values. In the case of binomial models, this graph shows a clear pattern in two lines. This is because the response variable can only take two possible values for each observation, and the predicted values are grouped around these two values.

## 3. Results

Descriptive characteristics of the sample are shown in Table 2.

Comparative analysis results of the characteristics of the sample according to the use of tracker devices to record daily activity (calories) are presented in Table 3.

GLM validation indicated no problems. The dispersion coefficient showed a value close to 1 (1.087) (Table 4); thus, no under or over dispersion of the data was detected.

**Figure 2:**
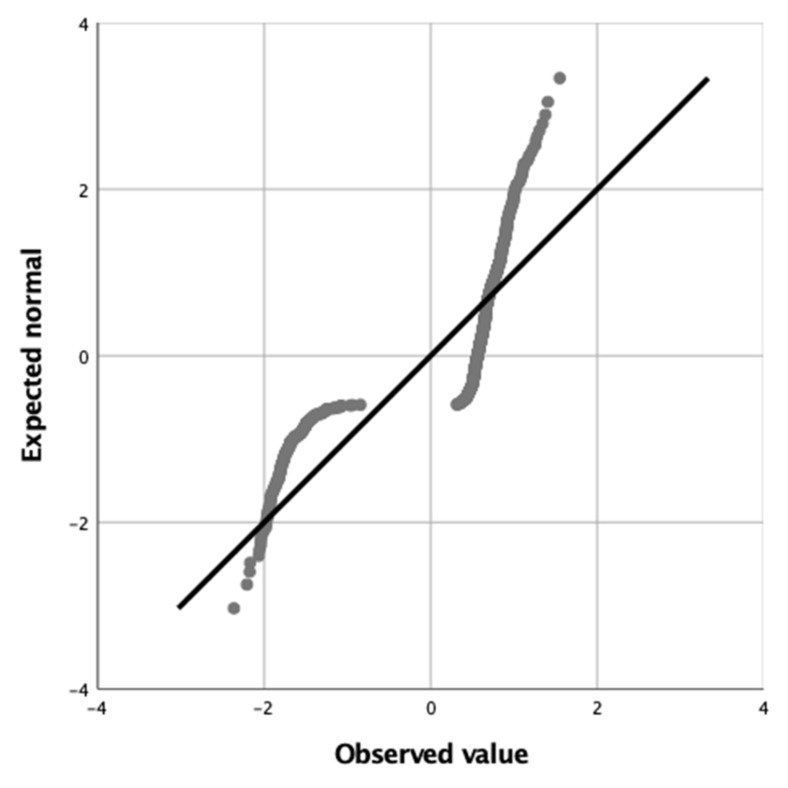
Probability plot showing the distribution of the deviance residuals.

The probability plot showing the distribution of the deviance residuals showed an adequate distribution with antisymmetric distal extremes and proximity of both medial extremes. A higher density of points was observed at the two medial extremes, located close to 0 (Figure 2).

**Figure 3:**
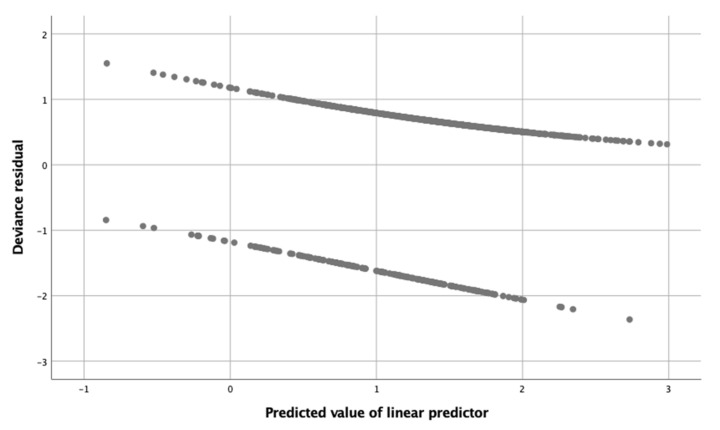
Scatterplot showing the relationship between deviance residuals and model predictions.

The relationship between deviance residuals and model predictions was verified by plotting deviance residuals versus predicted values. The scatterplot of residuals versus predicted values (Figure 3) showed a clear pattern because the response variable was binomial. A lower density of points was observed in the extremes corresponding to the highest absolute values of the deviance residuals.

The numerical outputs of the parameter estimates are given in Table 5. Significant effects for being female (*p* < 0.001), time spent sitting: low cardiovascular disease mortality risk (*p* = 0.001); time spent sitting: medium cardiovascular disease mortality risk (*p* = 0.023) and BMI (*p* = 0.007) were detected.

According to the estimate, being female increased the odds of having used a tracker device to record daily activity (calories) by 2.3 times in relation to being a male. Having a low cardiovascular disease mortality risk related to time spent sitting increased the odds of having used a tracker device to record daily activity (calories) by 2.7 times with respect to having a high cardiovascular disease mortality risk. Having a medium cardiovascular disease mortality risk related to time spent sitting increased the odds of having used a tracker device to record daily activity (calories) by 1.9 times with respect to having a high cardiovascular disease mortality risk. For every 1-point increase in the BMI score, the odds of having used a tracker device to record daily activity (calories) increased by 5.2%.

## 4. Discussion

Females and millennials had used more tracker devices to record daily activity. Fewer subjects who had ever used any tracker device to record daily activity had high or very high cardiovascular disease mortality risk due to the time spent sitting. Subjects who had ever used any tracker device to record daily activity had a higher BMI. The amount of vigorous or moderate physical activity or the time spent walking were not associated with the use of tracker devices to record daily activity.

Being female increased the odds of having used a tracker device to record daily activity by 2.3 times. To have a low or a medium cardiovascular disease mortality risk related to time spent sitting increased the odds of having used a tracker device to record daily activity with respect to having a high cardiovascular disease mortality risk. For every 1-point increase in the BMI score, the odds of having used a tracker device to record daily activity increased by 5.2%.

Little is known about how individual characteristics affect use of wearable activity trackers because existing research focuses mostly on the use associated with technical issues. In our sample, females had used more tracker devices to record daily activity, as it has been suggested previously on the web [32]. For this observed difference, it must be considered that females were predominantly represented in the sample. We did not find any previous manuscript that compared the use of tracker devices in adults according to gender. Gender differences represent an increasingly significant line of research because considering gender differences allows to make more precise recommendations and facilitates debate regarding its sociological implications [33]. According to this, previous research studied, in 2019, the perceptions of patients and family members regarding the acceptance of wearable devices as health tools. In general terms, they concluded that although men had a greater interest in wearable devices, the acceptance and use were also increasing in young women [34]. The results of our study seem to confirm this observation.

Generation refers to those individuals born in the same period or group of years, and who experienced the same or similar environmental, political, and social influences that would mold and impact that particular generation. These influences shape their beliefs, values, attitudes, and behavior [35]. The concept of generations is relevant as one of the main factors suggested to mold the reactions of a generation is technological advancement [36]. In our study, millennials were predominantly represented in the sample, and they had used many more tracker devices to record daily activity than the other generations in the sample; though this difference does not persist in the multivariate analysis, showing that there are factors related to the use of tracker devices that are more relevant than age. Only one previous study has shown that millennials are more prone to using m-health for lifestyle education but related to apps, and in a Malaysian sample, with more difficult access to new technologies than the sample of our study [37].

Subjects with low or a medium cardiovascular disease mortality risk related to time spent sitting in this study reported greater use of tracker devices to record daily activity. The use of Fitbits has previously been related to the interruption of sitting time of employees during sedentary work [38]. Sedentary behavior in diabetic adults has been related with impaired cardiometabolic health [39] and, independent of physical activity, with increased risk of diabetes, cardiovascular disease, and cardiovascular and all-cause mortality [40]. Replacing sedentary behavior with standing, sleeping (only with sleeping deficit), walking, and moderate to vigorous physical activity has been associated previously with mortality risk reductions [41]. It has been suggested that the deleterious effects of sedentary behavior are caused by a unique physiological pathway related to inactivity, considering that sitting too much is not the same as the lack of exercise and that sitting too much has its own metabolic consequences [42]. These metabolic consequences include: deep and fast descent in the concentration of plasma high-density lipoprotein cholesterol [43] and 90–95% loss of lipoprotein lipase activity, locally, in the most oxidative skeletal muscles in the legs, which is necessary for the uptake of fat from the blood so that it can be metabolized by muscle [44]. According to data of our and previous studies, we can conclude that the use of tracker devices can reduce the deleterious effects of sedentary behavior, promoting the decrease in time spent sitting, regardless of changes in physical activity.

The amount of vigorous or moderate physical activity or the time spent walking were not associated, in this study, with the use of tracker devices to record daily activity. A previous study with an adolescent sample has shown a positive association between the use of apps and wearable devices and the daily moderate-to-vigorous physical activity [45]. However, it has also been observed that tracker devices may act as facilitators, but they are not useful for health behavior change without any other intervention [46]. In fact, wearable activity tracker-based counseling intervention among patients with type 2 diabetes mellitus, overweight/obesity, cardiovascular diseases, chronic respiratory diseases, cognitive disorders, or sedentary older adults increased physical activity [47]. A recent review has shown that wearable devices are able to help with the control of diabetes, as well as prevent the complications associated with this condition [48].

Conversely to our results, recent reviews showed that the use of wearable activity trackers effectively improves the amount of physical activity, but not sedentary behavior [49,50]. Again, both reviews have included studies that compared interventions utilizing wearable activity trackers with interventions that do not utilize activity tracker feedback, and they did not analyze the use of wearable activity trackers in the general population.

In this study, higher BMI increased the odds of using a tracker device to record daily activity, which may be related to dissatisfaction with body weight. Although previous studies have achieved weight loss with short-term (<6 months) tracker device-based interventions [22]; it has also been reported that providing activity-level feedback alone, that is, the device alone, did not result in weight loss [51]. Recommending technological support alone, in programs for long-term weight loss purposes, is discouraged [52]. To be more effective, additional behavioral change techniques must be included, especially in individuals in whom dissatisfaction with body weight can generate anxiety regarding caloric intake. This is in line with the outcomes of Jakicic et al., who found that the addition of a wearable technology device, that provided feedback on energy expenditure, to a standard behavioral intervention resulted in less weight loss in healthy adults [53]. Otherwise, a positive association has also been shown between activity tracking frequency and weight loss [54].

The possible tracker devices used in this study were Fitbit, Apple Watch, Polar, Garmin, and Nike. These wireless devices are a useful tool for continuously monitoring physical activities and may assist patients suffering from chronic pathologies [55]. Thus, Fitbit, Apple Watch, and Garmin have been validated to examine heart rate and energy expenditure at different exercise intensities [56]. The highest measurement error has been found in heart rate for the three devices with respect to the Polar heart rate monitor in light and moderate physical activity, showing that the Apple Watch was the most accurate [56]. The evaluation of the devices regarding measurement of energy expenditure reported that the three devices measured more calories than the Parvo Medics TrueOne 2400 metabolic measurement system [56]. It has been concluded previously that most wrist-worn devices measure heart rate with an acceptable error but they poorly estimated energy expenditure [57]. In summary, wearable devices can help people to monitor their activity, but the potential measurement errors must be taken into consideration if they are used to follow health habits by healthcare professionals. However, the technology of wearable devices is constantly being improved, as well as the studies that validate their measurements, so we believe that they will increasingly become a more valid tool for following the health habits of the population with greater precision.

Clinical implications of these results are that tracker devices may be useful for promoting healthy habits, but with nuances. It seems that the use of wearables can modify habits such as excessive time spent sitting, unlike what happens with other habits that may require a more complex elaboration, such as carrying out a physical activity program or modifying the diet according to health standards. If the registered information of the devices was managed by healthcare professionals, it could provide wider benefits to users [7,58]. Health professionals can advise on behavioral change techniques and recommendations to guide their use. They may also assess the presence of adverse effects such as anxiety regarding monitoring or the presence of eating disorders, especially in persons with body dissatisfaction [14]. With the support of healthcare professionals, when necessary, tracker devices could make health self-management more achievable, positively affecting in the health of the population and the functioning of health systems, but the error measurement of the devices must be taken into consideration.

### Limitations

The present study has some limitations. It is limited by the cross-sectional design conducted in a sample using Instagram, with a predominance of females and millennials, and disseminated through universities. This can make it difficult to extrapolate the results to other samples less acquainted with the use of new technologies. The question in relation to tracker device use has not been validated previously. This question may have included more options for answering than yes/no, such as rarely, sometimes, and frequently to learn more about these trends, but the need to simplify the survey to facilitate its completion prevailed. Associations were examined, but no causal relationship can be established. It was discussed that people who have used tracker devices have reduced the time spent sitting, but it could also be interpreted that these subjects, more prone to taking care of their health, were less sedentary and they have resorted to using tracker devices to monitor their health status. The survey included only commercially available tracking devices to record daily activity (calories) and no other types of digital technologies, such as mobile applications.

## 5. Conclusions

Females have used tracker devices 2.3 times more than men. For every 1-point increase in the BMI score, the odds of having used a tracker device increased by 5.2%. Subjects with low cardiovascular disease mortality risk related to time spent sitting have used tracker devices 2.7 times more than subjects with high cardiovascular disease mortality risk. The amount of vigorous or moderate physical activity or the time spent walking were not associated with the usage of tracker devices to record the daily activity. These conclusions are limited by the cross-sectional design; thus, no causal relationship can be established. Future research should focus on testing the validity of the survey and the extension of the research to other population groups less acquainted with the use of new technologies, collecting more exhaustive data on the characteristics of the use of tracker devices.

## Figures and Tables

**Table 2:** Descriptive characteristics of the sample.

Characteristics		
Gender (n = 890)	n (%)	
Male	185 (20.8)	
Female	705 (79.2)	
**Generation (n = 892)**	**n (%)**	
Generation-Z (born 1997–2012)	103 (11.5)	
Millennials (born 1981–1996)	673 (75.4)	
Generation-X (born 1965–1980)	102 (11.4)	
Boomers (born 1946–1964)	14 (1.6)	
**Use of tracker device to record daily activity (calories) (n = 892)**	**n (%)**	
Ever used	687 (77)	
Never used	205 (23)	
**Time spent sitting (n = 892)**	**n (%)**	
Low cardiovascular disease mortality risk (n = 315)	315 (35.3)	
Medium cardiovascular disease mortality risk (n = 408)	408 (45.7)	
High cardiovascular disease mortality risk (n = 86)	86 (9.6)	
Very High cardiovascular disease mortality risk (n = 83)	83 (9.3)	
	**Mean**	**SD**
**Body Mass Index (n = 889)**	25.2	5.3
**Vigorous physical activity (min per week) (n = 762)**	297.9	283.5
**Moderate physical activity (min per week) (n = 736)**	321.8	417.5
**Time spent walking (min per week) (n = 843)**	812.2	1136.3

**Table 3:** Comparative analysis of the characteristics of the sample according to the use of tracker devices to record daily activity (calories).

	Use of Tracker Device to Record Daily Activity (Calories)		
	Ever Used	Never Used	*p* Value
**Gender (n = 890)**	**%**	**%**	
Male	17.6	68.5	<0.001
Female	82.4	31.5	
**Generation (n = 892)**	**%**	**%**	
Generation-Z (born 1997–2012)	9.5	18.5	0.001
Millennials (born 1981–1996)	78.6	64.9	
Generation-X (born 1965–1980)	10.5	14.6	
Boomers (born 1946–1964)	1.5	2.0	
**Time spent sitting (n = 892)**	**%**	**%**	
Low cardiovascular disease mortality risk (n = 315)	37.8	26.8	0.004
Medium cardiovascular disease mortality risk (n = 408)	45.3	47.3	
High cardiovascular disease mortality risk (n = 86)	9.0	11.7	
Very High cardiovascular disease mortality risk (n = 83)	7.9	14.1	
	**Mean (SD)**	**Mean (SD)**	
**Body Mass Index (n = 889)**	25.4 (5.3)	24.6 (5.0)	0.024
**Vigorous physical activity (min per week) (n = 762)**	304.1 (295.2)	274.8 (233.9)	0.291
**Moderate physical activity (min per week) (n = 736)**	327.4 (428.2)	301.6 (377.0)	0.510
**Time spent walking (min per week) (n = 843)**	831.9 (1156.9)	746.8 (1065.0)	0.470

**Table 4:** Generalized linear model. Goodness of fit.

	Value	Degrees of Freedom	Dispersion Coefficient
Deviance	810.132	745	1.087

**Table 5:** Generalized linear model. Parameter estimates.

Dependent Variable: Use of Tracker Device to Record Daily Activity (Calories)	Odds Ratio	Wald 95% Confidence Interval for the Odds Ratio. Lower Bound/Upper Bound.	Wald Chi-Square Statistic	*p* Value
Constant	0.197	0.036/1.069	3.546	0.060
Female	2.299	1.567/3.372	18.129	<0.001
Generation-Z (born 1997–2012)	0.677	0.188/2.439	0.356	0.551
Millennials (born 1981–1996)	1.632	0.479/5.556	0.615	0.433
Generation-X (born 1965–1980)	0.974	0.270/3.518	0.002	0.968
Time spent sitting: Low cardiovascular disease mortality risk	2.698	1.524/4.778	11.589	0.001
Time spent sitting: Medium cardiovascular disease mortality risk	1.870	1.090/3.211	5.161	0.023
Time spent sitting: High cardiovascular disease mortality risk	1.551	0.773/3.111	1.527	0.217
Body Mass Index	1.052	1.014/1.091	7.301	0.007

## Data Availability

The datasets analyzed during the current study are available from the corresponding author on reasonable request. All data analyzed during this study are included in this published article.
